# The Role of Magnetic Resonance Imaging in the Planning of Surgical Treatment of Deep Pelvic Endometriosis

**DOI:** 10.3389/fsurg.2022.944399

**Published:** 2022-06-28

**Authors:** Francesco Manti, Caterina Battaglia, Iennarella Bruno, Michele Ammendola, Giuseppe Navarra, Giuseppe Currò, Domenico Laganà

**Affiliations:** ^1^ Radiology Unit, Department of Experimental and Clinical Medicine, “Magna Graecia” University, Catanzaro, Italy; ^2^ Science of Health Department, Digestive Surgery Unit, University “Magna Graecia” Medical School, Catanzaro, Italy; ^3^ Department of Human Pathology of Adult and Evolutive Age, Surgical Oncology Division, “G. Martino” Hospital, University of Messina, Messina, Italy; ^4^ Science of Health Department, General Surgery Unit, University “Magna Graecia” Medical School, Catanzaro, Italy

**Keywords:** deep pelvic endometriosis, magnetic resonance imaging, diagnosis, surgical treatment, videolaparoscopic

## Abstract

**Background:**

To prospectively evaluate the diagnostic accuracy of magnetic resonance imaging (MRI) for the planning of surgical treatment of deep pelvic endometriosis.

**Materials and Methods:**

From January 2020 to December 2021, we evaluated 72 patients with symptoms characteristic of endometriosis to plan appropriate surgical treatment. Sensitivity (Se), specificity (Sp), positive and negative predictive values (VPP/VPN), and the accuracy of MRI for the detection of deep pelvic endometriosis were calculated.

**Results:**

Seventy-two patients (mean age, 35.5 years; range, 20–46 years) suspected of having pelvic endometriosis were recruited. Pelvic endometriosis was confirmed at pathologic examination in 56 (77.7%) of 72 patients. A total of 22 (39.3%) of 56 patients were subjected to video laparoscopy (VLS), and 16 (72.2%) of 22 were treated by surgery. Se, Sp, VPP, and VPN in intestinal endometriosis diagnosis were, respectively, 100%, 93.3%, 100%, and 87.5%, and diagnostic accuracy was 95.4%. MRI Se in ureteral endometriosis diagnosis was 50%, Sp 100%, VPP 100%, VPN 78%, and diagnostic accuracy 82%. MRI Se in endometrioma diagnosis was 92.3%, Sp 100%, VPP 100%, VPN 90%, and diagnostic accuracy 95.4%. MRI Se in rectum-vaginal septum (SRV) endometriosis diagnosis was 80%, Sp 100%, VPP 100% VPN 85.7%, and diagnostic accuracy 91%. The MRI Se in the diagnosis of endometriosis involving ULS was 100%, Sp 92.8%, VPP 89%, VPN 100%, and diagnostic accuracy 95.4%. Complete concordance results in a 100% accuracy for all calculated values in diagnosing bladder endometriosis localizations.

**Conclusion:**

MR imaging demonstrates high accuracy in detecting deep pelvic endometriosis in specific locations. It allows the localization of deep pelvic lesions with highly fibrotic components that are hardly recognizable with other imaging methods and not visible with VLS.

## Introduction

Endometriosis is a chronic inflammatory disease characterized by the presence and proliferation of endometrial stroma and glands in abnormal locations with a predilection for the ovaries, the pelvic viscera, and the peritoneum of the pelvic excavation (1, 2). Like normal uterine endometrium, ectopic endometrial mucosa responds to ovarian hormonal stimuli and, therefore, undergoes functional changes during the menstrual cycle (2, 3). Endometriosis can manifest in three primary forms: superficial endometriosis, ovarian endometrioma, and deep pelvic endometriosis (DPE) (3–7). Surgical treatment is a common approach for the diagnosis and treatment of endometriosis, particularly for patients non-responding to medical therapy and with severe symptoms (8). Because the surgical treatment may vary depending on the location, severity, and extent of involvement, accurate diagnosis and localization are crucial for appropriate management (9). In this regard, there are different types of surgeries based on localization. For example, for rectovaginal septum lesions, the surgical approach could be conservative and may include nodulectomy and shaving of the lesion or radical where the involved intestinal tract is resected. The surgical treatment of a patient with ureteral involvement can be conservative with ureterolysis or more aggressive with procedures such as ureterostomy or nephrectomy. There are two techniques for the surgical treatment of the bladder: transurethral resection (TUR) and partial cystectomy, which include segmental bladder resection (8). Techniques have proven necessary for correct disease staging and proper management (2). Today, video laparoscopy (VLS) has become a valuable tool for diagnosing and managing endometriosis. However, especially in cases associated with deep infiltrating lesions, MRI is a potentially helpful non-invasive imaging technique for precise localization and assessment of the extent of the disease before laparoscopic evaluation and surgical management. In this context, the goal of our study is to evaluate the diagnostic accuracy of MRI investigation in detecting deep endometriosis lesions for surgical treatment planning.

## Materials and Methods

From January 2020 to December 2021, we evaluated 72 patients with symptoms characteristic of endometriosis to plan an appropriate surgical treatment. All patients recruited in the study had, to varying degrees, distinct symptomatology for endometriosis (dysmenorrhea, dyspareunia, chronic pelvic pain, and infertility) and, in some cases, strongly suspect symptoms for intestinal endometriosis (diarrhea, constipation, rectal pain during the menstrual cycle, chronic pelvic pain, and dyschezia).

Given the symptoms and the objective examination, after a preliminary transvaginal ultrasound imaging, the patients were subjected to diagnostic completion with MRI to assess the presence and location of any endometriosis lesions to plan a proper therapeutic approach. MRI study predicts that on the day before the examination, patients make a preparation with a diet without slag and polyethyleneglycol (three sachets of Isocolan) dissolved in 1,500 ml of water (Macrogol 4000 29.500 g; sodium sulfate 2.843 g; sodium bicarbonate 0.843 g; sodium chloride 0.733 g; potassium chloride 0.371 g). MRI tests were performed with a 1.5 Tesla magnet (Achieva XR, Philips Medical System, Eindhoven, Netherland) using a four-channel surface coil (SENSE-body coil). The examination protocol included two phases: the first involved a resonance of the pelvis at high resolution (RM-HR), of a total duration of about 17 min, performed after distension of the vagina with 50–60 ml of sterile gel and administration of 10 mg intravenous *N*-Joscina butyl bromide (Buscopan, Boehringer Ingelheim, Milan, Italy), with the following sequences: turbo spin-eco (TSE) T2 weighted oriented in the sagittal plane, TSE T2 coronal, and T1 TSE axial. The T2 arrangements oriented in the para-axial plane, perpendicular to the long axis of the cervix, provided a circumferential view of the cervix and the upper floor of the vagina, which helped evaluate the involvement of the uterine-sacral ligaments. The second phase, Clisma-RM-CE, with a total duration of about 9 min, involved the distension of the colon, so the patients were placed in the left lateral decubitus on the table of the MRI to introduce a catheter of Foley (20 F) in the rectal and infuse 1,500 ml of PEG solution at 37 4 4 4, after pharmacological intestinal hypotonization by further administration of 10 mg iv of Joscina’s *N*-butyl bromide (Buscopan, Boehringer Ingelheim).

Once the bowel was fully distended, an intravenous injection of 0.1 ml/kg contrast agent paramagnetic Gadobutrol (Gadovist, Bayer Pharma AG, Müllerstrasse, Berlin, Germany) was administered. During post-processing, MIP reconstructions of urographic sequences (T1 VIBE coronal) were performed in all MRI investigations. MRI was well tolerated by all patients who completed the entire imaging protocol. In all cases, the images were adequate for diagnosis. The intestinal preparation was always sufficiently good. Out of 72 MRI patients, 22 were subjected to VLS. In the case of intestinal endometriosis involvement, the lesion’s presence, localization, and extent were evaluated. On the suspicion of pelvic ureter involvement, the localization, degree of involvement, ureteral dislocation (traction), and secondary extrinsic or intrinsic compression by the reactive fibrotic tissue were evaluated with MRI for the possible presence of hydronephrosis. Finally, other pelvic localizations of endometriosis (ovary, recto-vaginal septum, uterus-sacral ligaments, parametrium, and bladder) were also investigated. We calculated sensitivity (Se), specificity (Sp), positive predictive (VPP), and negative predictive (VPN) for the detection of deep pelvic endometriosis located in a different location.

## Results

Seventy-two patients (aged 20–46 years, average age of 35.5 years) were examined using MRI upon the clinical suspicion of endometriosis. A total of 56 patients (56/72) were found positive for endometriosis localization. Two patients (22/56) were subjected to VLP, and 16 (16/22) were treated by surgery. More precisely, 1 (1/16) patient was subjected exclusively to vitreolysis, 7 (7/16) patients with intestinal involvement were subjected, respectively, to colic resection (3/7), adhesives (2/7), and nodulectomy (2/7); 8 (8/16) patients with urethral participation underwent bilateral ureterolysis (3/8), monolateral ureterolysis (4/8), and urethral resection (1/8), respectively. We did not find lesions in the parametrium.

### MRI in Intestinal Endometrosic Lesions

**Figure 1:**
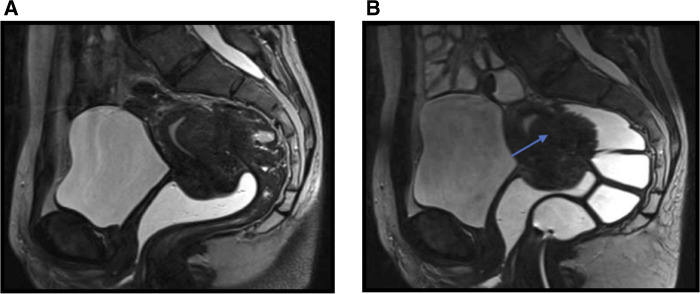
SAG TSE T2w, Intestinal involvement: (**A**) precolic distension, (**B**) postcolic distension with PEG; demonstrates a large endometrioma (thin arrow) adjacent to a recto-sigmoidal junction.

The MRI investigation identified eight intestinal lesions of endometriosis in eight patients, five in the rectum, two in the sigmoid-rectal passage, and one in the sigmoid. The laparoscopic investigation confirmed the presence of 7 intestinal endometriosis lesions in 7 patients treated with intestinal resection of the sigma-rectal tract in 3/7 cases, nodulectomy at the level of the rectum in 2/7 cases, and adhesiolysis in 2/7. One (1/8) distal sigmoid lesion reported to MRI was not confirmed to VLS (Figure 1).

MRI Se in intestinal endometriosis diagnosis was 100%, Sp 93.3%, VPP 87.5%, VPN 100%, and diagnostic accuracy 95.4% (Table 1).

**Table 1:** Results.

Place	Se (%)	Sp (%)	VPP (%)	VPN (%)	Accuracy (%)
Bowel	100	93.3	87.5	100	95.4
Ureters	50	100	100	78	82
Ovaries	91.6	90	91.6	90	91
SRV	80	100	100	85.7	91
USL	100	92.8		100	95.4
Bladder	100	100	100	100	100

*Se, sensitivity; Sp, specificity.*

**Table 2:** Personal casistic vs. literature.

	Clisma-RM		RM	
	Se (%)	Sp (%)	Se (%)	Sp
Bowel	100	93.3	94	97
Ureters	50	100	7	100
Endometriomas	92.3	100	86.3–97.1	73.6–91.3
SRV	80	100	44.4–84	77.8–100
ULS	100	92.8	56.4–93	61–96.5
Bladder	100	100	23.1–88	94.7–100

*Se, sensitivity; Sp, specificity.*

### MRI in Ureteral Endometrosic Lesions

**Figure 2:**
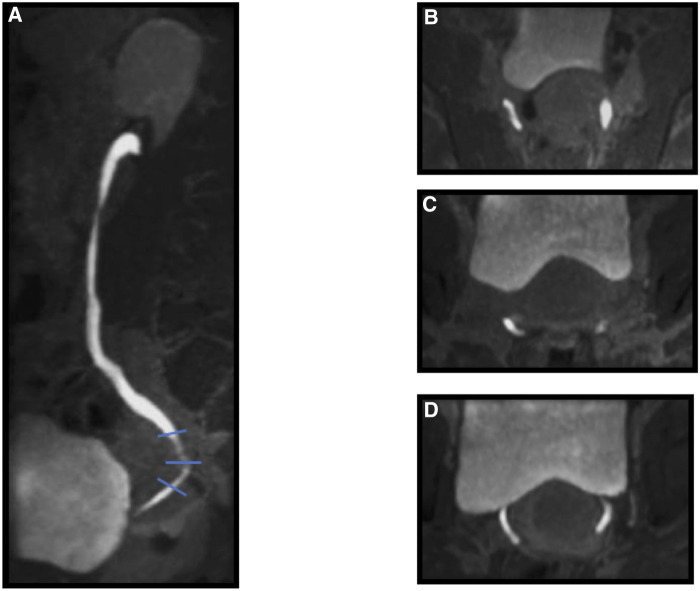
Ureteral involvement. T1 TSE – VIBE, postcontrast agent, urographic sequences: (**A**) SAG, (**B**) Para-AX: high level (thin line), (**C**) Para-AX: medium level (thin line), (**D**) Para-AX: inferior level (thin line).

The MRI investigation identified 5 ureteral lesions in 4 patients, the VLS 11 lesions in 8 patients, in 4/8, a monolateral ureterolysis was performed (in 2 of these also the excision of a periureteral nodule), and in 3/8, a bilateral ureterolysis was performed (in one of these also a nodulectomy). Finally, one (1/8) patient was subjected to ureteral resection and T-T anastomosis for intrinsic ureteral endometriosis. In all these cases, the MRI examination showed the late phase; in particular, in 2/4 positive points, MRI showed hydro-ureteronefrosis, and in the other two cases, ureteral dislocation angle without dilation was evident. The remaining 4 cases of ureteral involvement documented at VLS were not detected at MRI; of the three instances where VLS involvement was bilateral, in one case, MRI saw the participation of the left ureter, and in another place, there was a correct diagnosis of bilateral dislocation, and the other finally turned out a false negative (Figure 2).

MRI Se in ureteral endometriosis diagnosis was 50%, Sp was 100%, VPP was 100%, VPN was 78%, and diagnostic accuracy was 82% (Table 1).

### MRI in Ovarian Endometriosis

**Figure 3:**
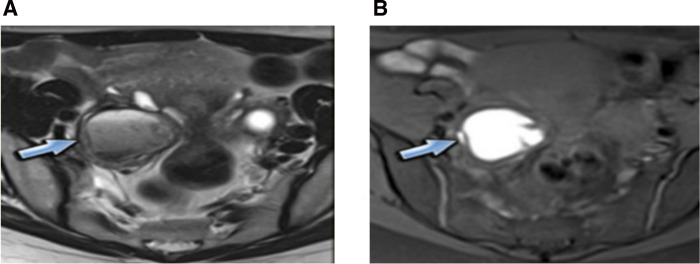
Ovary involvement: (**A**) T2w: “shading effect,” (**B**) T1w: hyperintensity.

Ovarian localizations of deep pelvic endometriosis were identified, including 17 ovarian endometriomas at MRI (4 left ovaries, 4 right ovaries, and 4 bilateral endometriomas) in 12 patients, at VLS, 17 endometriomas in 12 patients (5 right ovaries, 4 left ovaries, and 4 bilateral), only in 2/12. Patients were subjected to annessiectomy in a bilateral case and only one left (Figure 3). MRI Se in endometrioma diagnosis was 92.3%, Sp was 100%, VPP was 100%, VPN was 90%, and diagnostic accuracy was 95.4% (Table 1).

### MRI in Endometriosis Lesions of the Rectum-Vaginal Septum

**Figure 4:**
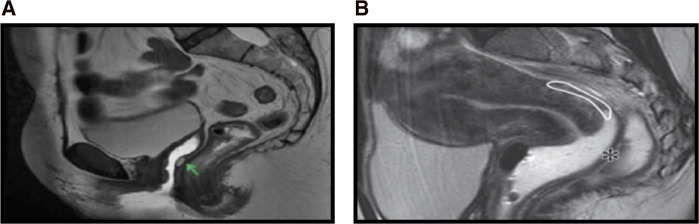
SAG T2: (**A**) prevaginal distension, (**B**) postvaginal distension.

The MRI also identified 8 nodules of the rectum-vaginal septum in 8 patients, surgical treatment, in this case, was excision of the nodule (Figure 4).

MRI Se in SRV endometriosis diagnosis was 80%, Sp 100%, VPP 100%, VPN 85.7%, and diagnostic accuracy 91% (Table 1).

### MRI in Endometriosis Lesions of Uterine-Sacral Ligaments

**Figure 5:**
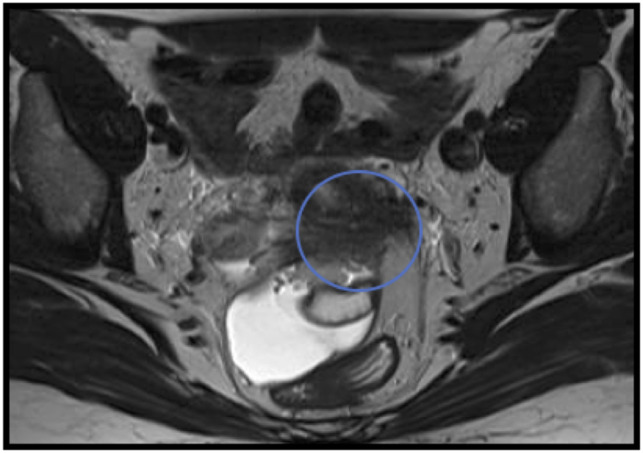
Para-AX T2, utero-sacral ligament involvement.

The MRI identified 15 lesions of the uterine-sacral ligaments in 9 patients, VLS 11 lesions in 8 patients; in this case, the removal of the nodules proceeded. (Figure 5). The MRI Se in the diagnosis of endometriosis involving ULS was 100%, Sp 92.8%, VPP 89%, VPN 100%, and diagnostic accuracy 95.4% (Table 1).

### MRI in Bladder Endometriosis Lesions

**Figure 6:**
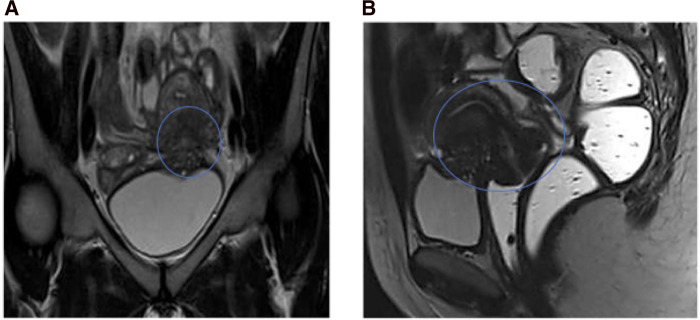
(**A**) bladder involvement, (**B**) visceral involvement.

The MRI finally identified two bladder lesions: in one case, it was a nodule of the upper bladder wall of 2 cm removed by partial cystectomy (Figure 6); in the other, essential adhesions were reported in the context of the vesicular fold uterine, corfirmated by the VLS, during which a complex adhesion process took place.

Complete concordance resulted in a 100% accuracy for all calculated values in diagnosing bladder endometriosis localizations (Table 1).

## Discussion

Several studies have shown that MRI is the second, after ultrasound, best non-invasive method for analyzing endometriosis, with an overall sensitivity, for different disease sites of 90% (10).

Particularly in the evaluation of ULS by MRI, Bazot et al., in a 2011 study, showed an increase in diagnostic accuracy compared with conventional MRI using an additional thin-layer para-axial T2 sequence (3 mm), which has a higher spatial resolution and better follows the anatomical course of the ULS (11). The RM-HR of the pelvis completed with intestinal distension (RM-enema) allows an accurate diagnosis of deep pelvic endometriosis, allowing, with a single examination and without the use of ionizing radiation, an evaluation of the appendages of the “cul de sac” posterior, anterior compartment, uterine-sacral ligaments, rectum-vaginal septum, parametrium, and ureteral and intestinal localizations. The latter can be better defined based on their mass-after-stress effect and the signal strength in the weighted T1 (with or without fat suppression) and T2 sequences.

The need to use paramagnetic MD remains uncertain as, according to some authors, it allows a more accurate assessment of the depth of infiltration of intestinal lesions and a more precise distinction between the rectal wall and the lesion; others claim that it does not represent added value (12, 13). Distension of the colon may be carried out with water or ultrasound gel (14, 15).

MRI with hydro distension of the colon and intravenous administration of paramagnetic contrast agent (RM-CE enema) allows a better “detection” of intestinal lesions, increasing diagnostic accuracy compared with pelvic MRI without distension, as reported in a study by Scardapane et al. (14).

Clisma-RM-CE, however, requires more execution time and maybe less tolerated by patients. Therefore, it is considered a diagnostic completion of pelvic RM-HR by these authors, which remains the preferred examination in the diagnosis of DPE, reserving colic distension to patients with a clinical suspicion of intestinal involvement or who, in the first stage of the examination, have severe deep pelvic lesions (14).

In this regard, from our experience, thanks to accurate informed consent and a comfortable clinical environment, all patients have been able to complete the entire examination protocol.

Vimeracti et al., in a 2012 study, also performed very well in evaluating intestinal and ureteral endometriosis with RM-CE enema with values of Se, Sp, VPP, and VPN of 100% (13).

According to Flaxman et al., for the surgical plan of endometriosis, the 3D reconstruction of 2D dimensional images taken with MRI will be the new way for surgeons to improve anatomical comprehension with a better vision of structural orientation, but unfortunately, in our study, we did not use this handy tool (16).

In our series were included 22 patients subjected to enema-RM-CE for the diagnosis of deep pelvic endometriosis and compared with the VLS, considered the gold-standard reference. As for the intestinal lesions, we evaluated the presence, the seat, and the extent but not the degree of trans-parietal infiltration. The RM-CE enema examination in our personal experience showed sensitivity and specificity values in the diagnosis of intestinal endometriosis of 100% and 93%, respectively, 3% of which were sufficiently consistent with those obtained in similar studies of Scardapane et al. corresponding to 94% and 97%, respectively (14). The MRI examination was also optimized for studying the excretion via uro-MRI sequences (T1 coronal VIBE in the late phase with subsequent MIP reconstructions).

In our case study, 4/8 cases of ureteral involvement were identified, while the remaining 4 cases were falsely negative. As all these were subjected to ureterolysis and only one to colectomy in the absence of hydro-ureteronefrosis, it can be hypothesized that the lack of diagnosis was due to the presence of an extrinsic involvement that did not determine pathological conditions recognizable to MRI.

The Se and Sp values obtained were 50% and 100%, respectively, which for sensitivity was at variance with the literature whose values were 75% and in perfect agreement with the Sp (100%) (17). Our study shows that the diagnosis of ureteral endometriosis by MRI presents a wide margin for improvement, assuming that it is always necessary to carry out the excretion phase for a correct study.

As for the other localizations affected by deep pelvic endometriosis, there is no doubt that the MRI results are more accurate than CT in their detection.

Proof of this is that Biscaldi et al., in a 2014 study, compared the two methods (MDTC-e and RM-e) in reference only to the rectum localizations. The authors justify this choice by stating that the comparison of the identification of the other deep pelvic localizations is not feasible, because MRI, in this context, represents the “gold standard.” CT is not used for this purpose because of the low contrast resolution inherent in the method, which generates poor diagnostic accuracy for pelvic localizations of the disease (18). A comparison with the laparoscopic finding showed that MRI could detect lesions in ovaries with an SE of 92.3% and an Sp of 100 (if 86.3%–97.1% and Sp 73.6%–86.8%) (13, 19, 20).

Comparing our case studies and with the data reported in the literature, it was found that the sensitivity and specificity in the detection of endometriosis lesions at the rectovaginal septum were perfectly average: If 80% vs. 44.4–84%, Sp 89% vs. 77.8–100% (13, 14, 17, 21–23). Scanning SRV lesions in the sagittal plane preceded vaginal distension with an ultrasound gel. With regard to lesions of the uterine-sacral ligaments, our results do not differ significantly from those of other studies conducted previously: Se 100% vs. 56.4%–93% and Sp 92.8% vs 61%–96.5% (11–14, 17, 18, 21).

In this context, the thin-layer T2 para-axial sequences that better support the anatomy of the ligaments have been able to achieve a significant improvement in diagnostic accuracy.

Our results in the study of the bladder were 100% sensitivity and specificity; in particular, the latter was in line with the results of other studies (Sp 94.7%–100%), while the sensitivity was significantly higher than that found in the literature (23.1%–88%). However, the low incidence of bladder involvement in our case studies (2/22 cases) (12, 13, 17, 20–24) is to be noted (Table 2). The main limitations of our study were represented by the reduced number of patients subjected to VLS after performing MRI, failure to assess the degree of parietal infiltration of the colon, and finally, the absence of a comparison with the results of histology.

## Conclusion

The MRI is the most accurate method in the study of pelvic endometriosis. It is considered the most critical examination in the planning of patients to undergo laparoscopic or laparotomic surgery.

MRI, in fact, non-invasively and without the use of ionizing radiation, provides a panoramic evaluation of the pelvis and, thanks to the high contrast resolution, allows to characterize deep pelvic lesions with a high fibrotic component hardly recognizable with other imaging methods and not visible to the VLS as sub-peritoneal. The completion of the examination with MRI-enema, moreover, allows to reach high values of diagnostic accuracy for lesions involving the intestine; in fact, for a correct localization/measurement of colic lesions, the water distension of the colon is necessary. It is essential to have the excretion phase accompanied by MIP reconstructions in evaluating the ureters. However, our study in this field shows some limits, especially for the diagnosis of extrinsic lesions (adhesions) that do not affect hydro-ureteronefrosis. Another limitation was the absence of 3D reconstruction. With good inter-operator reproducibility, RM-CE enema investigation is proposed in preoperative planning to ensure the best treatment approach and address the surgical choice between nodulectomy or segmental colic resection, ureterolysis, or ureteral resection. However, the proposed protocol requires intestinal preparation and implies a longer investigation time to reduce patient compliance. In this regard, with our experience in the study of deep pelvic endometriosis, we conclude that multidisciplinary assessment, a comfortable clinical environment, a fully informed consent regarding the modalities of the MRI investigation, and the diagnostic advantages of this approach form the basis of a full collaboration of patients and have allowed obtaining excellent diagnostic results.

## Data Availability

The raw data supporting the conclusions of this article will be made available by the authors, without undue reservation.
